# Load-bearing biodegradable PCL-PGA-beta TCP scaffolds for bone tissue regeneration

**DOI:** 10.1002/jbm.b.34691

**Published:** 2020-08-04

**Authors:** Alok Kumar, Mohammad Mir, Ibrahim Aldulijan, Agrim Mahajan, Aneela Anwar, Carlos H. Leon, Amalia Terracciano, Xiao Zhao, Tsan-Liang Su, Dilhan M. Kalyon, Sangamesh G. Kumbar, Xiaojun Yu

**Affiliations:** 1 Department of Biomedical Engineering, Stevens Institute of Technology, Hoboken, New Jersey; 2 Department of Chemical Engineering and Materials Science, Stevens Institute of Technology, Hoboken, New Jersey; 3 Department of Civil, Environmental and Ocean Engineering, Center for Environmental Systems, Stevens Institute of Technology, Hoboken, New Jersey; 4 Department of Biomedical Engineering, University of Connecticut, Storrs, Connecticut; 5 Department of Orthopedic Surgery, University of Connecticut Health, Farmington, Connecticut; 6 Department of Biochemistry and Molecular Biology, School of Medicine, University of Maryland, Baltimore, MD; 7 Department of Basic Sciences and Humanities, University of Engineering and Technology, Lahore, Pakistan

**Keywords:** bone, mechanical properties, PCL, PGA, TCP

## Abstract

A biocompatible and biodegradable scaffold with load-bearing ability is required to enhance the repair of bone defects by facilitating the attachment, and proliferation of cells, and vascularization during new bone formation. However, it is challenging to maintain the porosity and biodegradability, as well as mechanical properties (especially compressive strength), at the same time. Therefore, in the present work, a biodegradable composite structure of poly(caprolactone) (PCL) was designed using compression molding with varying amounts of poly(glycolic acid) (PGA) (25, 50, 75 wt%) and fixed amount (20 wt%) of beta tricalcium phosphate (beta TCP). It was hypothesized that the fabricated composite structure will develop porosity during the degradation of the PGA and that the corresponding decrease in mechanical properties will be compensated by new bone formation and ingrowth, in vivo. Accordingly, we have systematically studied the effects of sample composition on time-dependent dissolution and mechanical properties of the PGA/beta TCP scaffolds. The compressive strength increased up to ~92 MPa at 50% compression of the designed PCL-PGA samples. Furthermore, the dissolution rate, as well as weight loss, was observed to increase with an increase in the PGA amount in PCL. Based on the mechanical properties and dissolution data, it is concluded that the PCL-PGA scaffolds with beta TCP can be suitable candidates for bone tissue engineering applications, specifically for the reconstruction of bone defects, where strength and biodegradation are both important characteristics.

## 1 | INTRODUCTION

Orthopedic injuries have always been one of the important focus areas of medicinal research. Natural bone has the tendency to heal by itself as far as small injuries are concerned.^1^ However, in the case of major fractures (segmental bone defects), the body is unable to heal the damaged bone on its own completely. In such cases, the size of the damaged area is sufficiently large to stop the bone from regenerating itself on its own (critical-sized defects) and frequently leading to the formation of pseudoarthrosis (false joint/nonunion). A critical-sized defect is considered to occur when the defect length is greater than ~2.5 times the diameter of the affected bone.^2^ Furthermore, the anatomic location of the defect and the state of the soft tissues surrounding the defect site also greatly impact the healing process.

In general, bone allografts and autografts are transplanted to augment and accelerate the process of bone regeneration, with autologous bone grafting constituting the gold standard for the healing of segmental bone defects. The possible dangers of disease transmission, serious infections with allografts, in addition to poor osteoconductivity and relatively poor mechanical strength, restrict the wide-scale applications of allografts.^3^ The application of autograft is limited by the requirement of prolonged anesthetic periods for harvesting, restricted availability, donor site morbidity, extended hospitalization, the peril of infection, and the tendency to fail due to dissimilar properties.^4,5^

During the last two decades, various innovative biomaterials were developed for bone tissue regeneration.^6–11^ Such biomaterials provide alternative approaches and offer great promise in the promotion of bone healing and regeneration. The latest research has focused on either the use of porous implants “scaffolds” or tissue-engineered biological constructs obtained by seeding bioactive scaffolds with cells, that is, the “tissue constructs.” The establishment of scaffold-based bone engineering has been a key development in the field of orthopedics.^12,13^

For adequate bone regeneration, the implanted scaffolds or scaffold-based tissue constructs must furnish adequate mechanical strength and firmness to make up for the lost mechanical function of the damaged tissues. The firmness and strength of the implanted scaffolds or tissue constructs should be sufficiently high to provide adequate structural support and permit the transmission of regeneration augmenting forces to the host tissue location. Cell turnover and tissue remodeling are essential to attain stable biomechanical conditions and vascularization, which are greatly impacted by the properties of scaffolds that include structural integrity, controlled porosity, sufficient mechanical strength, and biodegradability.^14–17^

Biodegradable synthetic polymers, such as poly(lactic acid) (PLA), poly(caprolactone) (PCL) poly(glycolic acid) (PGA), and their copolymer poly(lactide-co-glycolide) (PLGA), are well established for use as scaffolds in various clinical applications.^18–21^ PCL exhibits good mechanical properties and biocompatibility with a slower degradation rate than other polymers.^19^ On the other hand, PGA is a hydrophilic biodegradable polymer that allows the retention of the mechanical strength of scaffolds constructed from it.^22–24^ Thus, the use of a fast degrading hydrophilic polymer with a slow degrading polymer can be a model biomaterial for the bone tissue engineering application, which provides biodegradability as well as adequate mechanical strength during biodegradation during bone regeneration in vivo.

In the current study, composites of PCL and PGA were compounded with beta tricalcium phosphate (beta TCP). The beta TCP is an extensively used CaP ceramic in bone tissue engineering applications due to its excellent biocompatibility and osteoconductivity.^25–27^

The present work aims to produce a biodegradable composite scaffold with an initial compressive strength, that is, comparable to the cortical bone with intended use in the reconstruction of bone defects. After providing initial stability to the defect area, the faster degradation of hydrophilic polymer (e.g., PGA) leaves a porous scaffold of slow degrading polymer (e.g., PCL), available for vascularization and bone ingrowth. It is hypothesized that the decrease in strength will be compensated by the newly formed bone.

## 2 | EXPERIMENTAL PROCEDURE

### 2.1 | Materials

PCL (Cat. No. 440744, MW: 80,000), PGA (Cat. No. 06525, MW: >100,000), and beta TCP powder (Cat. No. 440744) were procured from Sigma Aldrich, Polysciences, and Fluka, respectively. An organic solvent, 1,1,1,3,3,3-Hexafluoro-2-propanol (HFIP, Cat. No. 003409) was purchased from the Oakwood Chemicals.

### 2.2 | Scaffold fabrication

Porous hybrid scaffolds of PCL-PGA-beta TCP were prepared using the solvent casting method, followed by compression molding and sintering. At least three compositions, based on the weight percent ratio were selected: 25PCL-75PGA-20beta TCP (P75), 50PCL-50PGA-20beta TCP (P50), 75PCL-25PGA-20beta TCP (P25).

**FIGURE 1:**
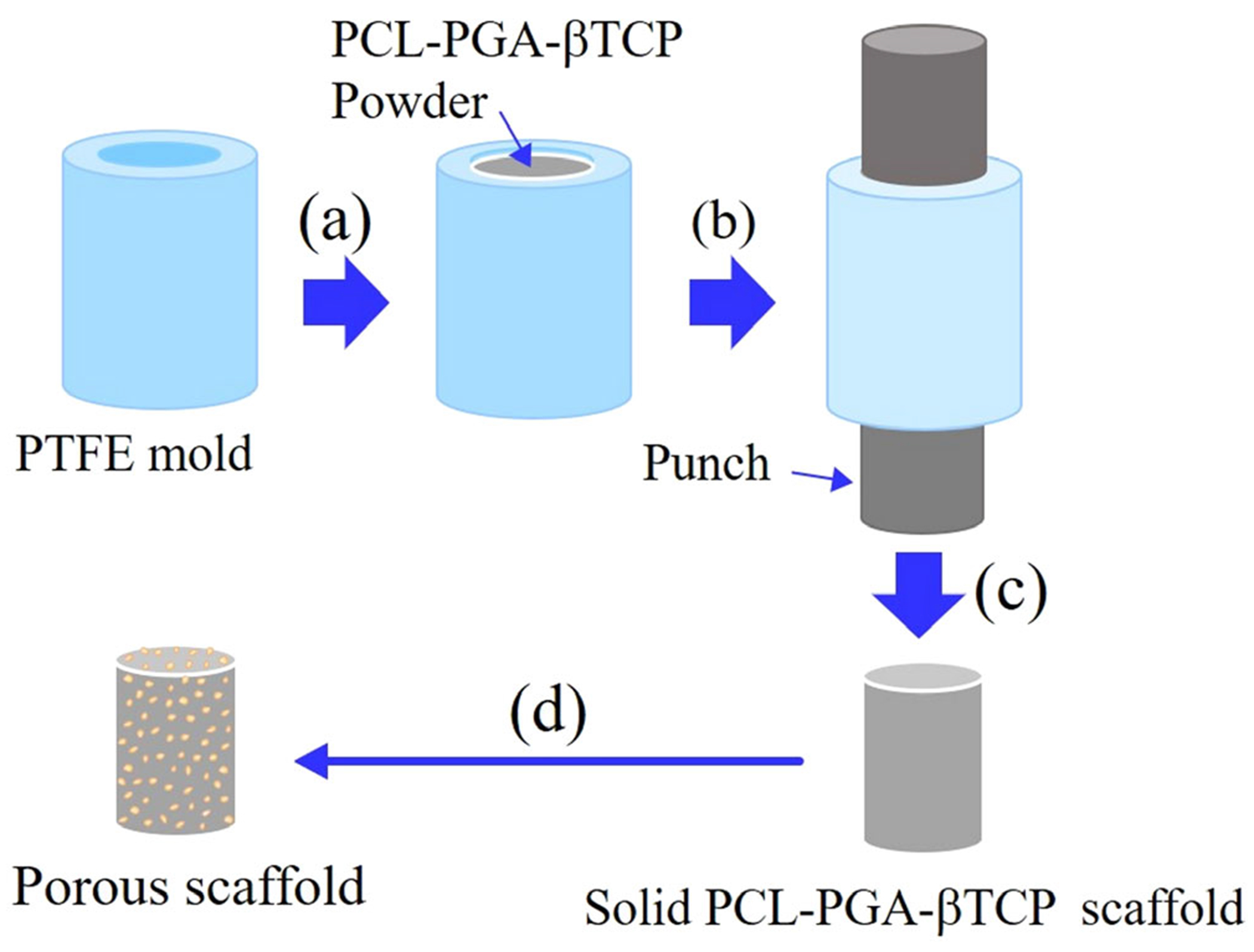
Schematic showing the fabrication of PCL-PGA-beta TCP scaffolds using compression molding of precursor PCL-PGA-beta TCP powder, followed by sintering. (a) PCL-PGA-beta TCP composite powder was transferred to the cylindrical-shaped mold. To prepare the PCL-PGA-beta TCP powder, PCL, PGA, and beta TCP were mixed in HFIP, followed by drying at room temperature. The dried powder was treated with liquid nitrogen and then crushed into a fine powder. (b) Mold was heated to 150°C and samples was compressed to 50% of the initial height. (c) The samples were reheated to 150°C to relieve the stress generation due to compression, followed by cooling. (d) During the in vitro dissolution study in 1×PBS, faster degradation of PGA leads to the creation of a porous PCL scaffold. beta TCP, beta tricalcium phosphate; HFIP, 1,1,1,3,3,3-Hexafluoro-2-propanol; PCL, poly(caprolactone); PGA, poly(glycolic acid)

PCL and PGA are both soluble in the highly fluorinated organic solvent, HFIP. Thus, HFIP was used as a solvent to make the starting materials for the PGA-based hybrid samples: Px (x = 75, 50, 25). Due to the corrosive and volatile nature of HFIP, the entire process was conducted within a fume hood. First, PGA was added gradually into HFIP, and the mixture was agitated with a magnetic stirrer for 48 hr, followed by the gradual addition of beta TCP and further stirring for 24 hr. Finally, PCL was added into the PCA/beta TCP/HFIP mixture, which was stirred for 24 hr. The resulting mixture was freeze-dried and then treated with liquid nitrogen to make powder. The powder of PCL-PGA-beta TCP was transferred to a cylindrical-shaped (6 mm diameter, 25 mm height) PTFE (Teflon) mold followed by heating in air at 150°C and then furnace cooling (Figure 1). Subsequently, samples were compressed to 50% of the initial sample size. These samples were further heated to relieve the stresses generated during compression, followed by furnace cooling. The molded porous samples were polished using SiC emery paper to remove surface irregularities.

All the prepared samples were washed in 100% ethanol for three times, 2 min each, followed by drying at room temperature. The dried samples were stored at room temperature in a desiccator until further characterization.

### 2.3 | Phase and microstructural characterization

For the microstructural characterization, sintered samples were gold coated (EM MED020, Leica, Germany) and then imaged under a scanning electron microscope (SEM, Auriga 40, Zeiss, Germany), operated at an accelerating voltage of 10 kV. Energy dispersive spectroscopy (EDS, X-Max, Oxford Instruments, UK) was used for the elemental analysis to assess the distribution of beta TCP in the sample.

X-ray diffraction (XRD) of as-sintered samples was carried out to study the phase transformation, if any, during sintering. For this, an Ultima IV (Rigaku, USA) was operated at 40 kV, and 30 mA with Cu K_α_ radiation (*λ* = 0.15418 nm) was used in the 2θ range of 20–60° and step size of 0.02°. The data were collected at an incident angle of 5° and using a scanning rate of 2°/min. The obtained XRD data were compared with the International Center for Diffraction Data (ICDD) and the existing literature for the phase analysis.

### 2.4 | Density and contact angle

The density and percentage porosity of sintered samples were analyzed using a liquid (ethanol) displacement method. For this, the dried sample (with weight W) was immersed in a graduated cylinder containing *V*_1_ volume of 100% ethanol. After a series of brief evacuation–repressurizations, the volume of ethanol (*V*_2_) was recorded. Following this, the volume of residual ethanol in the measuring cylinder was noted (*V*_3_) after the removal of the sample from the ethanol. This leads to the estimation of the volume of the sample without pores (*V*_2_ – *V*_1_) as well as pore volume (*V*_1_ – *V*_3_). Therefore, density (*d*) and percentage porosity of the sample can be expressed as *W/V* where *V* (=*V*_2_ – *V*_3_) is the volume of the specimen, and ([*V*_1_ – *V*_3_]/*V*) × 100, respectively.

Sintered samples were further characterized to study the effects of composition on wettability by deionized water via contact angle measurements. For this, a goniometer was used to measure the static contact angle of deionized water (using the sessile drop method) on smooth surfaces of 25PCL-75PGA-20beta TCP, 50PCL-50PGA-20beta TCP, and 75PCL-25PGA-20beta TCP samples.

### 2.5 | Mechanical properties

The sintered samples were tested under compression loading to measure the elastic modulus, 0.2% yield strength, and compressive strength at a speed of 1.3 mm/min. For the testing, cylindrical samples of 6 mm diameter, 12 mm height were prepared. A 5980 Instron universal testing machine was used in the compression mode. Compression data were collected following a 50% deformation (a 50% reduction of the initial sample height).

### 2.6 | Scaffold stability in aqueous conditions

For sterilization, the sintered samples were washed in 100% ethanol and then air-dried, followed by UV irradiation for 30 min. After measuring their weights, cylindrical samples of ~3 mm height and ~ 6mm diameter were kept in 4 mL 1×PBS under stirring at 37°C for 2, 4, 6, 8, 16, and 24 weeks. After dissolution, the samples were removed and gently washed in deionized water three times for 2 min. These samples were stored at −80°C, followed by freeze-drying. The weight and dimensions of the dried samples were measured, and the data were used to calculate the volume. Furthermore, Equation (1) was used to calculate the change in porosity due to dissolution:

(1) $$\text{Percentage change in porosity=}\left[\left(V_{i}-V_{f}\right)/V_{i}\right]\times 100,$$

where *V*_i_ = total volume of the sample before dissolution and *V*_f_ = total volume of the sample after the dissolution.

## 3 | RESULTS

### 3.1 | Scaffolds

As-sintered samples provided cylindrical samples with smooth surfaces, with no visible macropores. The density values of P75, P50, and P25 samples were ~ 1.5, ~ 1.9, and ~ 1.6 g/cm^3^, respectively. Furthermore, the measured contact angles of deionized water on P75, P50, and P25 were 71.4 ± 2.7, 74.5 ± 0.4, and 81.8 ± 0.9, respectively, indicating that the samples were not completely hydrophilic for water.

### 3.2 | Microstructure and phase assemblage

**FIGURE 2:**
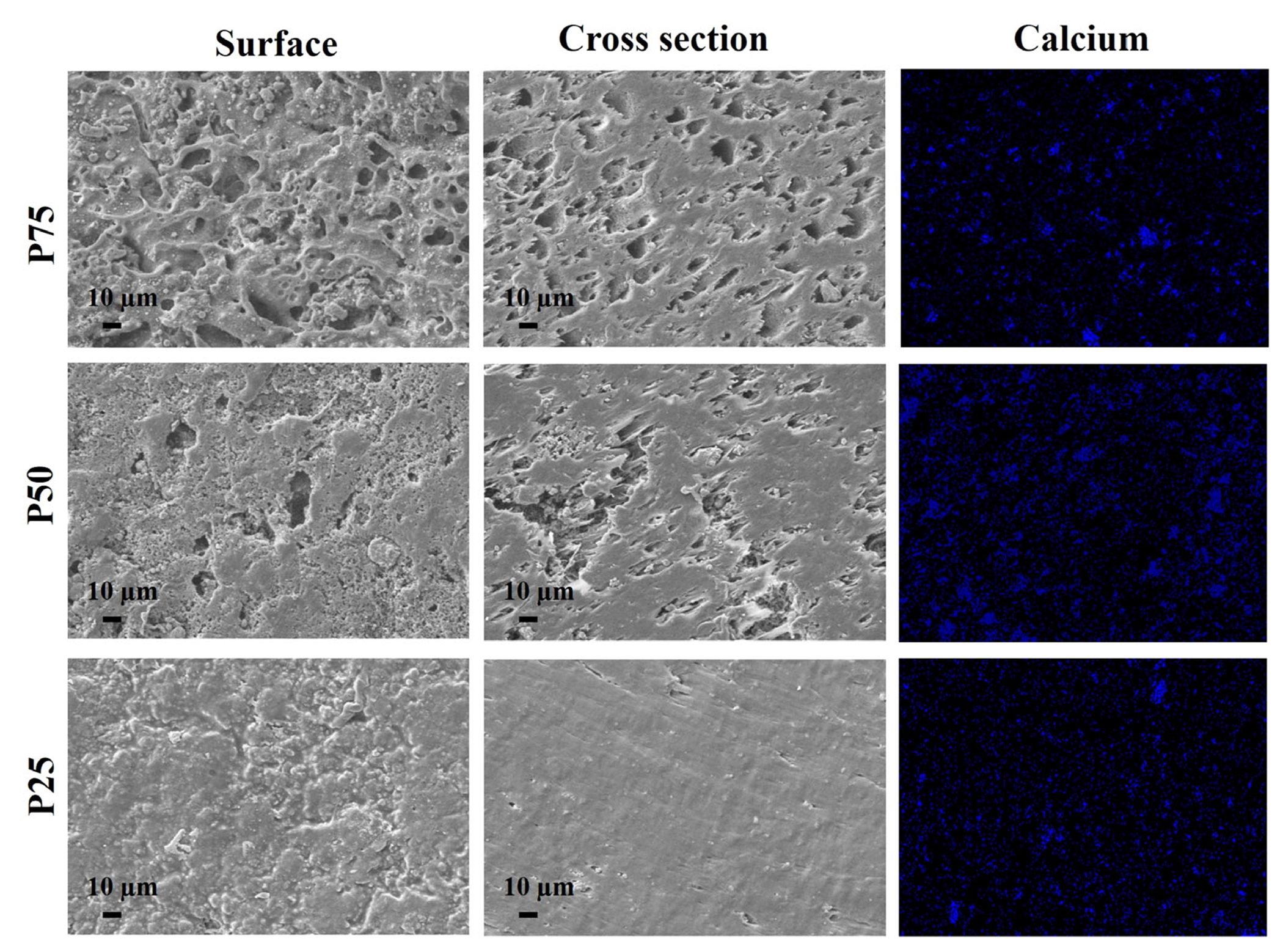
The representative scanning electron microscopy images of sintered 25PCL-75PGA-20beta TCP (P75), 50PCL-50PGA-20beta TCP (P50), and 75PCL-25PGA-20beta TCP (P25) samples. Results in secondary electron mode showing the presence of pores in P75 and P50 samples. However, no pores are visible in case of P25 sample. The EDS mapping data is showing the uniform distribution of calcium and phosphorous in all three samples, which confirm the presence of beta TCP and its uniform distribution in all PCL-PGA samples. beta TCP, beta tricalcium phosphate; EDS, Energy dispersive spectroscopy; PCL, poly(caprolactone); PGA, poly(glycolic acid)

As sintered P75, P50, and P25 samples were studied under secondary electron mode using SEM. The SEM micrographs revealed rough topographies with higher porosity in higher PGA contained samples (Figure 2). SEM of a cross-section of samples further confirmed the presence of pores inside the samples in the case of P75 and P50. However, no significant porosity was found in the case of P25. Importantly, P75 was characterized by more pores than P50.

**FIGURE 3:**
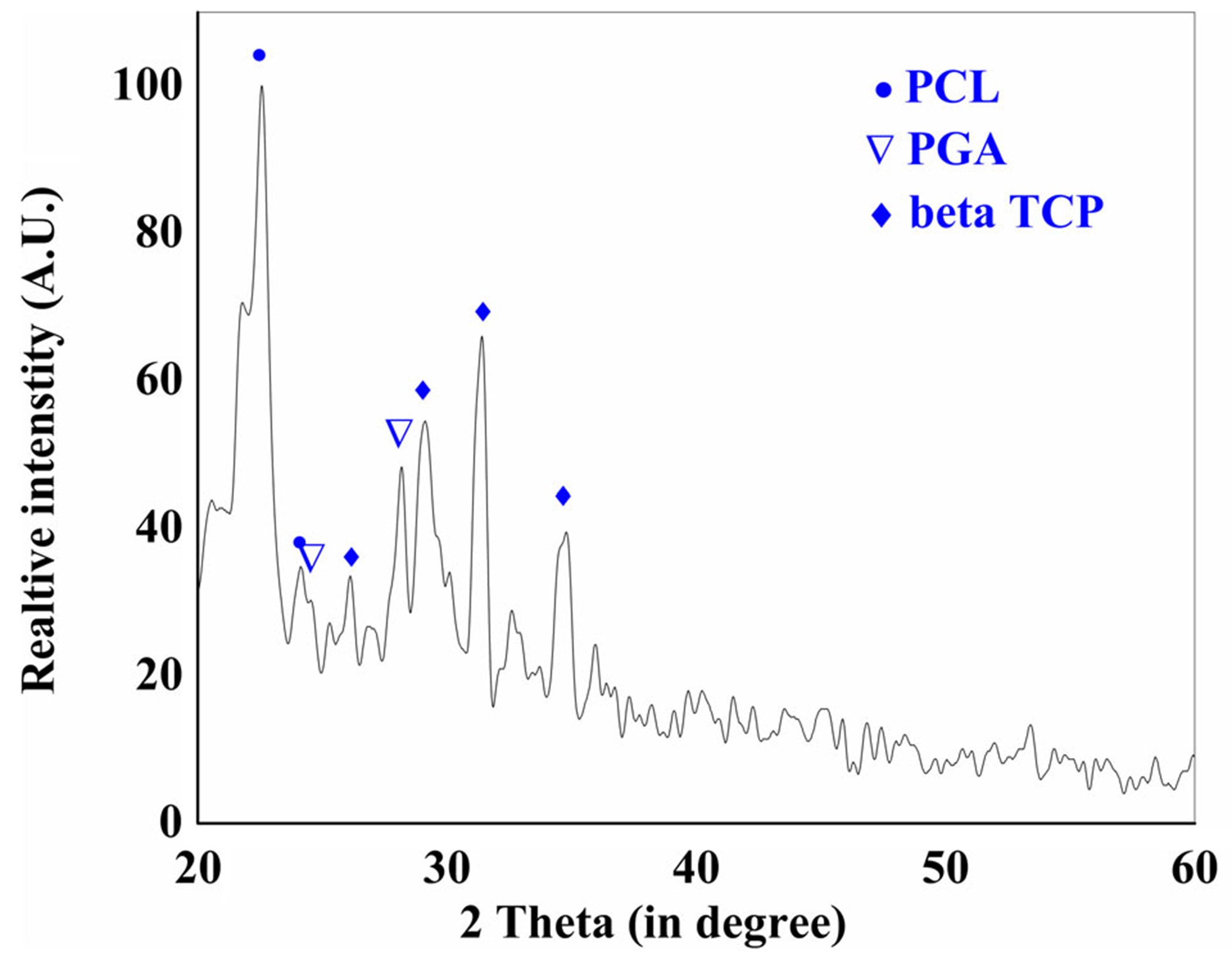
The representative X-ray diffraction of 50PCL-50PGA-20beta TCP (P50) sample, showing the presence of no phases other than PCL, PGA, and beta TCP in the sintered samples. The analysis confirmed the crystalline nature of beta TCP as well as semi-crystalline nature of PCL and PGA. beta TCP, beta tricalcium phosphate; PCL, poly(caprolactone); PGA, poly(glycolic acid)

The EDS mapping confirmed the presence of calcium and their homogenous distribution in all PCL-PGA-beta TCP samples, which is an indicator of beta TCP presence (Figure 2). A comparison of XRD pattern with ICDD database (for beta TCP, pdf # 32–0176) and the existing literature confirmed the presence of beta TCP, PCL,^28^ and PGA^29^ in the samples (Figure 3). The peaks associated with beta TCP, PCL, and PGA were marked with solid diamond, solid circle, an inverted triangle, respectively.

### 3.3 | Mechanical properties

**FIGURE 4:**
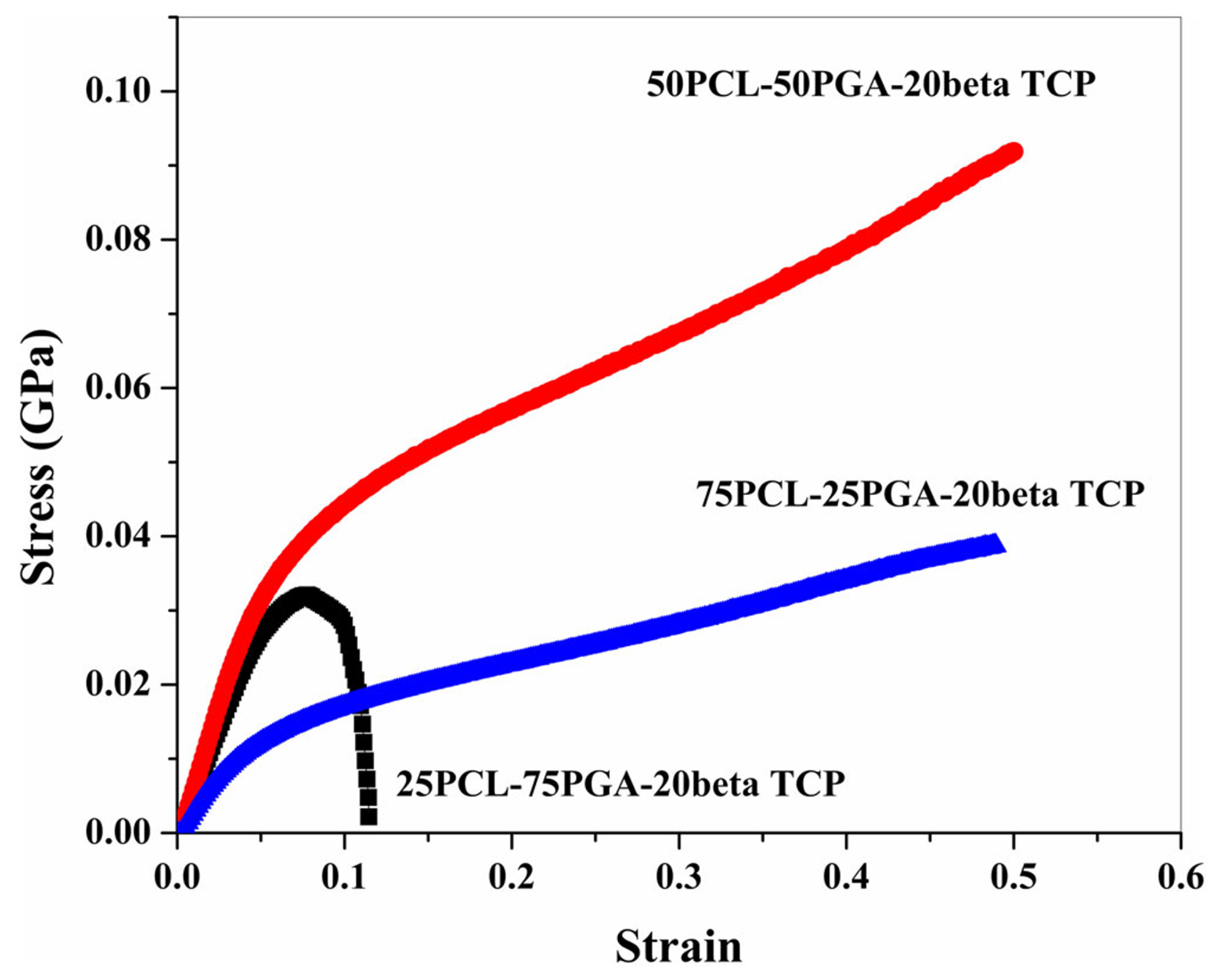
The representative compressive testing data showing the deformation behavior of 25PCL-75PGA-20beta TCP (P75), 50PCL-50PGA-20beta TCP (P50), and 75PCL-25PGA-20beta TCP (P25) samples at room temperature at a compression rate of 1.3 mm/min. Results showed a brittle behavior of high PGA content samples (P75) as compared to samples with lower PGA content (P50 and P25). As compared to the compositions used in this study, the samples with an equal amount of PCL and PGA showed the highest compressive strength. beta TCP, beta tricalcium phosphate; PCL, poly(caprolactone); PGA, poly(glycolic acid)

Figure 4 (and Supplementary Figure 1) shows the representative compression testing data of sintered P75, P50, and P25 samples. Results showed a significant increase in compressive properties, including the stress at yield, the stress at 50% deformation, and Young’s modulus with an increase in PGA concentration in PCL-PGA composite, going from 25 to 50% PGA. However, when the PGA concentration in the polymer mixture increased to 75%, a significant drop in mechanical properties ensued. Especially the toughness of the specimens was observed to decrease. The factors that may play a role on this include the possible decrease of the wettability of the beta TCP particles by the binder blend at the highest concentration of the PGA in the binder blend, which can, in turn, affect the mixing dynamics and prevent the proper encapsulation of individual TCP particles by the PLC/PGA binder blend. The effects of mixing/encapsulation of the solid filler particles by the polymeric binders are well documented in the literature but were not probed here.^30–32^ The calculated value of 0.2% compressive stress at yield for the P75, P50, and P25 samples were 29 ± 3, 26.3 ± 1.2, and 7.25 ± 0.75 MPa, respectively. The compressive strength at 50% deformation values were 26.5 ± 1.5, 92.33 ± 0.33, and 30± 9 MPa for P75, P50, and P25, respectively. For 25PCL-75PGA samples, compressive strength was lower than the yield strength. However, for 50PCL-50PGA and 75PCL-25PGA samples, compressive strengths were higher than the yield strength. The values of Young’s modulus were 0.58 ± 0.01, 0.70 ± 0.06, and 0.30 ± 0.01 GPa for the P75, P50, and P25 samples, respectively. Results showed an increasing trend in the yield strength and modulus with an increase of the PGA concentration. In the case of P75, with a higher amount of PGA than PCL, the compressive stress–strain curve showed an increasing trend in compressive stress during loading until ~32 MPa, and then it decreased with further increase in stress. The crack was initiated from the upper end of the samples, which leads to the fracture. Under compressive loading, samples with an equal amount of PCL and PGA showed an entirely distinct behavior with a prominent effect of PCL on deformation. An extensive deformation in the sample was noted with no sign of a surface crack. A higher value of compressive strength than yield strength was observed in this case. In the case of P25 samples in which the amount of PGA was lower than the PCL, the plastic deformation in the samples was observed at a much lower load than P75 and P50. As compared to a low value of yield strength (~7 MPa), samples showed a higher value of compressive strength.

### 3.4 | Stability of scaffolds in the aqueous conditions

**FIGURE 5:**
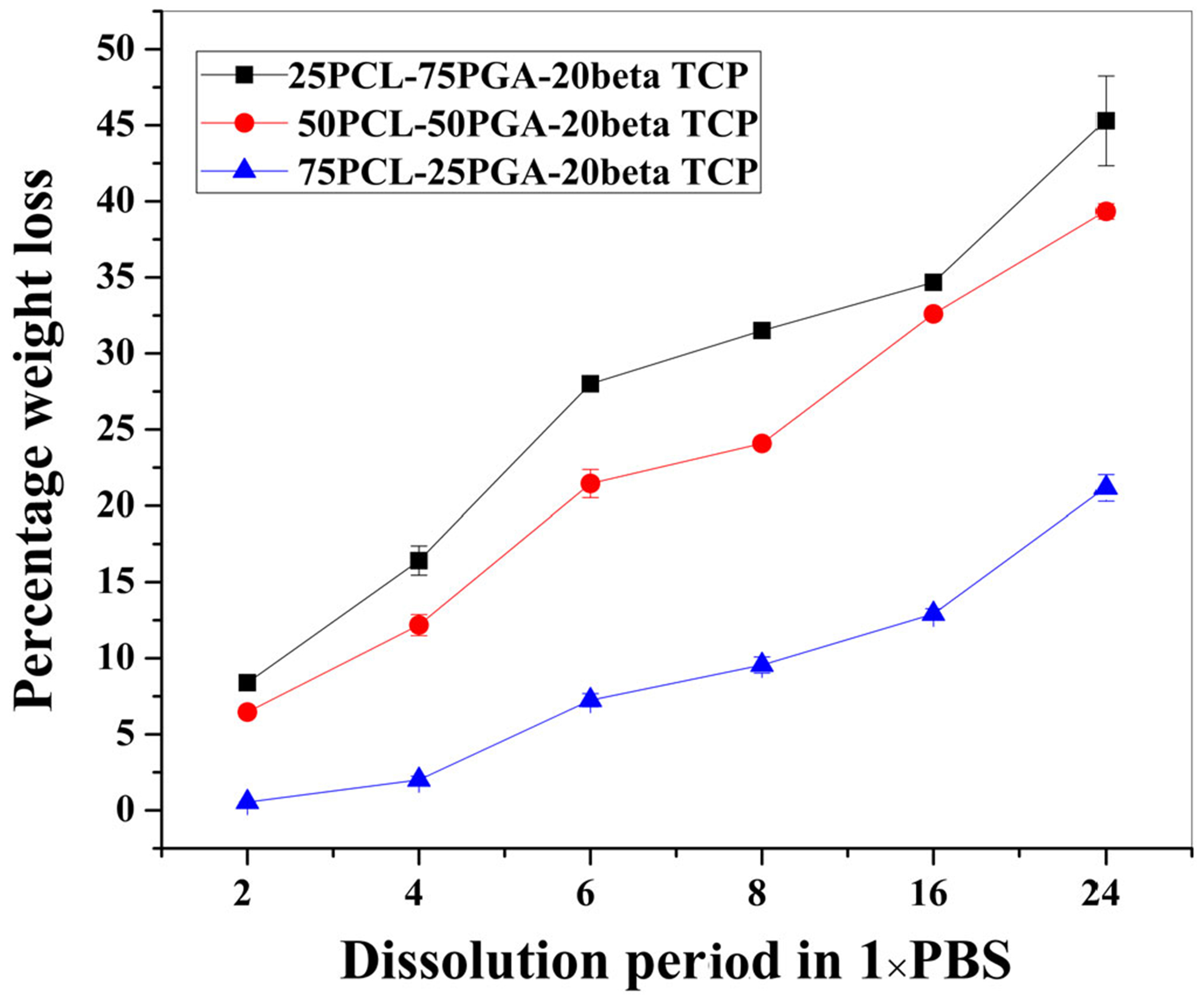
The dissolution data showing the time-dependent degradation of 25PCL-75PGA-20beta TCP (P75), 50PCL-50PGA-20beta TCP (P50), and 75PCL-25PGA-20beta TCP (P25) samples at 37°C temperature and in rocking condition. Results showed a decrease in composite weight due to the degradation of PGA and removal from the PCL matrix. Furthermore, a higher rate of dissolution was noted in the case of samples with a higher amount of PGA. beta TCP, beta tricalcium phosphate; PCL, poly(caprolactone); PGA, poly(glycolic acid)

Figure 5 shows the results of the dissolution study in 1×PBS at 37°C under dynamic (rocking) conditions. Figure 5 shows an approximately linear trend in percentage weight loss in all three compositions, P75, P50, and P25, as a function of dissolution time with correlation coefficient (*R*^2^) values .95, .90, and .82, respectively. A determining role of PGA was found on the weight loss. A higher weight loss was observed in the samples with a higher amount of PGA. An increase in PGA amount in PCL from 25 to 50 wt% leads to 6% increase in weight loss (0–6%) on 2 weeks, 10% (2–12%) on 4 weeks, 14% (7–21%) on 6 weeks, 14% (10–24%) on 8 weeks, 19.5% (13–32.5%) on 16 weeks, and 18.1% (21.3–39.4%) on 24 weeks. In contrast, an increase in PGA from 50 to 75 wt.% leads to 2.5% increase in weight loss (68.5%) on 2 weeks, 4.4% (12–16.4%) on 4 weeks, 7% (21–28%) on 6 weeks, 7.5% (24–31.5%) on 8 weeks, 2.1% (32.5–34.6%) on 16 weeks, and 5.5% (39.4–45.1%) on 24 weeks.

## 4 | DISCUSSION

In the context of biodegradability, polymeric scaffolds have significant advantages over metals and ceramics. However, with most polymeric materials, the compressive strength of the implant is lower than native bone tissue, which renders most polymers unsuitable for some orthopedic applications. Another factor that needs to be considered for the choice of materials for implants is vascularization and bone regeneration subsequent to implantation. For vascularization and bone generation, porous structures with interconnected pores are required in the scaffold.^33^ These interconnected pore channels help in the efficient transfer of oxygen and nutrients to the cells inside the porous scaffolds. However, a porous structure can be achieved only at the cost of the compressive strength of a scaffold. Higher porosity results in the reduction of the compressive strength.^34^ Thus, in the present study, a composite material of PCL and PGA with beta TCP as a bioactive additive was designed to provide porosity with interconnected pores as well as an acceptable mechanical property upon implantation. The blend of PCL and PGA with beta TCP was compressed and sintered to fabricate a densified scaffold with the capability to generate interconnected pores after the implantation. As noted earlier, the degradation rate of PGA is higher than the dissolution rate of PCL (Figure 1 and Figure 5). The absence of pores in the scaffold at the point of implantation ensures obtaining relatively high compressive properties of the scaffold and therefore, guarantee the implant stability during the early stage of implantation.

The faster degradation of PGA (2–5 months^35^) than PCL, which can take up to 1 year,^36^ results in the formation of a porous PCL/beta TCP structure. The formation of pore channels due to degradation of PGA should help in the neovascularization of the scaffold, that is, implanted and, therefore, enables the efficient supply of oxygen and nutrients to the cells as a porous structure develops with time upon the degradation of PGA.^37^ The volumetric loss in scaffold due to dissolved PGA is anticipated to be compensated by the new bone formation and ingrowth. Furthermore, the release of the osteoconductive beta TCP at the host site due to degradation of the PGA phase of the scaffold will help in the bone mineralization and faster recovery.

The adopted processing method was found suitable in the fabrication of PCL-PGA-beta TCP scaffolds for orthopedic applications without affecting the composition. The XRD data confirmed the presence of all three phases, PCL, PGA, and beta TCP in the sintered samples. The presence of beta TCP was further supported by the EDS data, which confirmed the uniform of distribution of beta TCP phase (Ca and P) in the matrix of PCL-PGA. The presence of a ceramic phase (beta TCP) is expected to help in the improvement in the compressive strength of the samples.

A strong effect of PGA on the physical properties of samples was noted. Results confirmed the density of designed samples (P75 ~ 1.3, P50 ~ 0.77, and P25 ~ 0.84 g/cm^3^) in the range of cancellous bone (0.1–1.0 g/cm^3^).^38^ Regardless of relatively high porosity, high PGA containing samples exhibited higher density values (Figure 2). This is due to a relatively higher density of PGA (~1.723 g/cm^3^)^39^ in comparison to the density of PCL (~1.135 g/cm^3^).^40^ We noted the distinct effect of PCL and PGA on the young modulus, compressive strength, and yield strength. PGA is known for its higher elastic modulus in comparison to that of PCL. This results in a higher elastic modulus in P50 than P25. However, a lower elastic modulus in P75 than P50 is related to the highly porous structure of the as-prepared P75 samples (Figure 2).

A higher yield strength in high PGA content samples can be generally correlated with a higher glass transition temperature. The glass transition temperature of PGA (35–45°C) is above the room temperature and allows high PGA content samples to withstand higher loads before the initiation of plastic deformation under room temperature conditions. Also, bulk PGA is characterized by a significantly higher compressive strength (~200 MPa)^41^ than PCL (~39 MPa).^42^ Despite high glass transition temperature and compressive strength (as reported in the literature), samples with high PGA content (P75) exhibited a sudden fracture after 10% compression, showing a compressive strength lower than P50 and P25 samples. This rapid loss in mechanical stability can be related to the high porosity and brittle behavior of P75 samples.

The PCL is known for its glass transition temperature much lower than the room temperature (−60°C) and, therefore, shows ductility during the deformation at room temperature. High PCL content samples (P25) showed higher compressive strength than low PCL content samples (P75) at 50% compression. A higher compressive strength was associated with the welding of pores (increases the density of the sample) during the loading as well as an increase in the cross-sectional area without cracking at the later stage of deformation.

Interestingly, higher compressive strength in the case of P50 (50PCL-50PGA) is related to the combined effect of PCL and PGA mechanical properties. Since beta TCP is equally added in all three compositions, therefore, to make the explanation simple, we are not considering the effect of TCP in the data analysis. PGA is expected to resist the plastic deformation, while PCL believes in preventing crack propagation during compressive loading.

Time-dependent degradation of the designed scaffold is very important to supply the beta TCP in the surrounding area as well as to support the bone ingrowth required to compensate for the mass loss due to scaffold degradation in vivo.^33^ The faster degradation of PGA leads to the formation of a porous scaffold, which may allow the blood vessels to grow inside scaffold.^43^ A clear effect of PGA on the dissolution was found with a higher rate of degradation in higher PGA samples (Figure 5). Degradation in 1×PBS showed a linear trend in weight loss. Importantly, a linear trend is important to accurately predict the time of the complete dissolution of the scaffold, which is required in the designing of the most appropriate implant for an application.

The P50 samples showed a weight loss and dissolution rate comparable to P75, but much higher compressive strength. Based on the current study, P50 (50PCL-50PGA scaffolds with 20 wt% beta TCP) were found suitable for the repair of bone defects due to high compressive strength (~92 MPa) which is much higher than cancellous bone (7–10 MPa) but lower than cortical bone (170–193 MPa).^38^

Although designed compositions have mechanical properties comparable to cancellous bone, further improvement in the compressive strength and elastic modulus is required to match the properties of a cortical bone. In the future, further improvement in the mechanical properties can be achieved by making a more densified scaffold by adopting the mechanical mixing of PCL, PGA, and beta TCP, followed by extrusion. Additionally, the degradation rate can be tailored by optimizing the wettability of the scaffolds in 1×PBS.

## 5 | CONCLUSIONS

The present study reports the biodegradable composites of PCL and PGA with beta TCP for the reconstruction of bone defects. The addition of PGA in the PCL matrix helped in the improvement in the compressive strength with a value higher than the cancellous bone. Furthermore, the addition of PGA results in the increase in the dissolution rate of PCL-PGA-beta TCP composites in 1×PBS. P75 and P50 samples were characterized by high porosity as compared to P25. Furthermore, the formation of pores during the dissolution is expected to help in the blood vessel ingrowth, which will help in the new bone formation and faster repair of damaged bone.

## Supplementary Material

Supplementary Material

Additional supporting information may be found online in the Supporting Information section at the end of this article.
