# Supplementary material for: Load-bearing biodegradable PCL-PGA-beta TCP scaffolds for bone tissue regeneration
Source: J Biomed Mater Res B Appl Biomater. Author manuscript; Available in PMC 2026 Apr 6. (PMC13051343; doi:10.1002/jbm.b.34691)
Supplement: Supplementary Material [file NIHMS2157381-supplement-Supplementary_Material.docx]

**Supplementary Material**

**Table S1.** Thermal properties of PGA blends with PCL at different ratios

| Polymer and their blends | $T_{m}$, $^{\circ}C$ | ${\Delta H}_{m}$, J/g | $T_{c}$, $^{\circ}C$ | ${\Delta H}_{C}$, J/g | $T_{g}$, $^{\circ}C$ | ${\Delta H}_{m}^{^{\circ}}$, J/g | $X_{c}$, % |
| --- | --- | --- | --- | --- | --- | --- | --- |
| PGA  PCL | 223.2  56.6 | 62.4  51 | 176.2  20.3 | 67.4  58.2 | 47.4  -65.4 | 183.2  139.5 | 34%  36% |
| PGA: PCL (25:75)  PGA  PCL | 221.3  55.9 | 77.1  59.2 | 190.3  27.5 | 6  56.6 | -65.1  (single) | 183.2  139.5 | 42%  42.4% |
| PGA:PCL (50:50)  PGA  PCL | 222.9  55.5 | 55.4  51.77 | 191.2  26.2 | 75  51.9 | -65  (single) | 183.2  139.5 | 30.2%  37% |
| PGA:PCL (75:25)  PGA  PCL | 217.8  53.1 | 45.5  19.5 | 187.3  23.1 | 87.8  35.7 | ND | 183.2  139.5 | 24%  13% |

**Table S2.** Characteristic FTIR bands for pure PCL

| **Band position (cm^-1^)** | **Description of vibrations** | **Reference** |
| --- | --- | --- |
| 2935 | Symmetric CH_2_ stretching | (*61*) |
| 2847 | Asymmetric CH_2_ stretching | (*61*) |
| 1722 | C=O stretching | (*61, 62*) |
| 1470, 1361 | CH_2_ bending | (*62*) |
| 1158 | C-O stretching | (*62*) |
| 1294 | C-C stretching | (*61, 62*) |
| 1047 | C-O-C stretching | (*61*) |

**Table S3.** Characteristic FTIR bands for pure PGA

| **Band position (cm^-1^)** | **Description of vibrations** | **Reference** |
| --- | --- | --- |
| 2991 | C-H stretching | (*63*) |
| 1741 | C=O stretching | (*63*) |
| 1418 | COO, C-H | (*63*) |
| 1148 | C-O stretching | (*64*) |
| 1081 | C-O-C stretching | (*63, 64*) |
